# A novel *EYA4* mutation causing hearing loss in a Chinese DFNA family and genotype-phenotype review of *EYA4* in deafness

**DOI:** 10.1186/s12967-015-0483-3

**Published:** 2015-05-12

**Authors:** Aiping Huang, Yongyi Yuan, Yanping Liu, Qingwen Zhu, Pu Dai

**Affiliations:** Department of Otolaryngology, Head & Neck Surgery, The Second Hospital Of Hebei Medical University, Heping West Road No.215, Shijiazhuang, Hebei 050018 China; Department of Otolaryngology, Head & Neck Surgery, Chinese PLA General Hospital, Fuxing Road No.28, Beijing, 100853 China; Department of Otolaryngology, Head & Neck Surgery, Children’s Hospital Of Hebei Province, Jianhua South Street No.133, Shijiazhuang, Hebei 050030 China

**Keywords:** *EYA4*, DFNA10, Hearing loss, Phenotype, Next-generation sequencing

## Abstract

**Background:**

Hereditary hearing loss is a heterogeneous class of disorders showing various patterns of inheritance and involving many genes. Mutations in the *EYA4* gene are responsible for postlingual, progressive, autosomal dominant hearing loss at the DFNA10 locus.

**Methods:**

We report on a Chinese family with sensorineural, progressive hearing loss. Next-generation sequencing (NGS) was conducted using DNA samples from this family. A candidate mutation was confirmed by Sanger sequencing. A detailed genotype and phenotype analysis of *EYA4* in deafness is provided.

**Results:**

NGS revealed an insertion mutation c.544_545insA in exon 8 of *EYA4* in all affected family members. This insertion created a frameshift resulting in a stop codon at position 221 (p.F221X). The p.F221X frameshift mutation cosegregated with hearing loss in the family. Audiograms of affected family members are flat or sloping, differing from the characteristic “cookie bite” audiogram and the mutation is localized in a different region of the eyaHR in *EYA4*.

**Conclusions:**

We identified a novel frameshift mutation in the *EYA4* gene. Our results enrich the mutational spectrum of *EYA4* and highlight the complexity of the DFNA10 genotypes and phenotypes. Using NGS techniques to establish a database of common mutations in patients with hearing loss and further data accumulation will contribute to the early diagnosis and development of fundamental therapies for hereditary hearing loss.

## Introduction

Hearing loss is one of the most common perception disorders and a highly heterogeneous sensory disorder. Non-syndromic hearing loss has both recessive and dominant modes of inheritance. It is estimated that 80% of the genetic forms of hearing loss are autosomal recessive and the remaining 20% are autosomal dominant [1]. Autosomal dominant non-syndromic hearing loss (ADNSHL) is characterized by heterogeneous genetic and clinical features. To date, more than 60 loci for ADNSHL have been mapped, but only 30 corresponding genes have so far been identified (http://hereditaryhearingloss.org).

“Classical” genetic studies such as linkage analyses, are expensive and time-consuming, and it has been difficult to sequence the hundreds of genes in the candidate chromosomal loci. Recently, high-throughput sequencing, also known as next-generation sequencing (NGS) has been shown to be an ideal tool to overcome these limitations through its ability to perform parallel sequencing of millions of nucleotides at relatively low cost and high speed. The capacity to simultaneously screen thousands of target genes makes this technique an especially powerful tool for detecting pathogenic mutations that cause heterogeneous disorders such as hereditary hearing loss [2].

**Figure 1:**
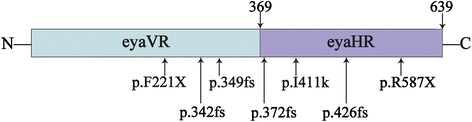
The general structure of *EYA4* protein showing the positions of the identified mutation in DFNA10 families (arrows). (N, N-terminus, C, C-terminus, eyaHR, eya-homologous region, eyaVR, eya-variable region) [5].

Among ADNSHL genes, *EYA4* was first identified as a causal gene at DFNA10 by O’Neill in a large American family in 1996 [3]. All Eya proteins contain a highly conserved 271-amino-acid C-terminal eya-homologous region (eyaHR) and an N-terminal proline-serine-threonine (PST)-rich transactivation domain known as the eya-variable region (eyaVR) (Figure 1). The eyaHR is essential for members of the EYA protein family in terms of their interaction with PAX, SIX, and DACH proteins in a genetic network that is conserved across species [4,5]. “Eyes absent 4” (*EYA4*), a member of the vertebrate *Eya* family of transcriptional activators, is a causative gene of postlingual, progressive, autosomal dominant hearing loss at the DFNA10 locus.

To date, six families at the DFNA10 locus have been identified. Almost all reported cases show a similar phenotype, characterized by postlingual, progressive, “cookie bite” tonal audiometric curves. In this study, we examined the genetic basis of ADSHNL in a Chinese family by NGS and identified a novel mutation, p.F221X, in exon 8 of the *EYA4* gene. The age of onset and audiograms of the affected members in the family also differ from those in previous reports.

## Materials and methods

All procedures were approved by the Ethics Reviewing Committee of the Second Hospital of Hebei Medical University as well as the Chinese PLA General Hospital. The study was carried out only after written informed consent was obtained from each individual and/or the parents of minors.

### Family and clinical evaluation

**Figure 2:**
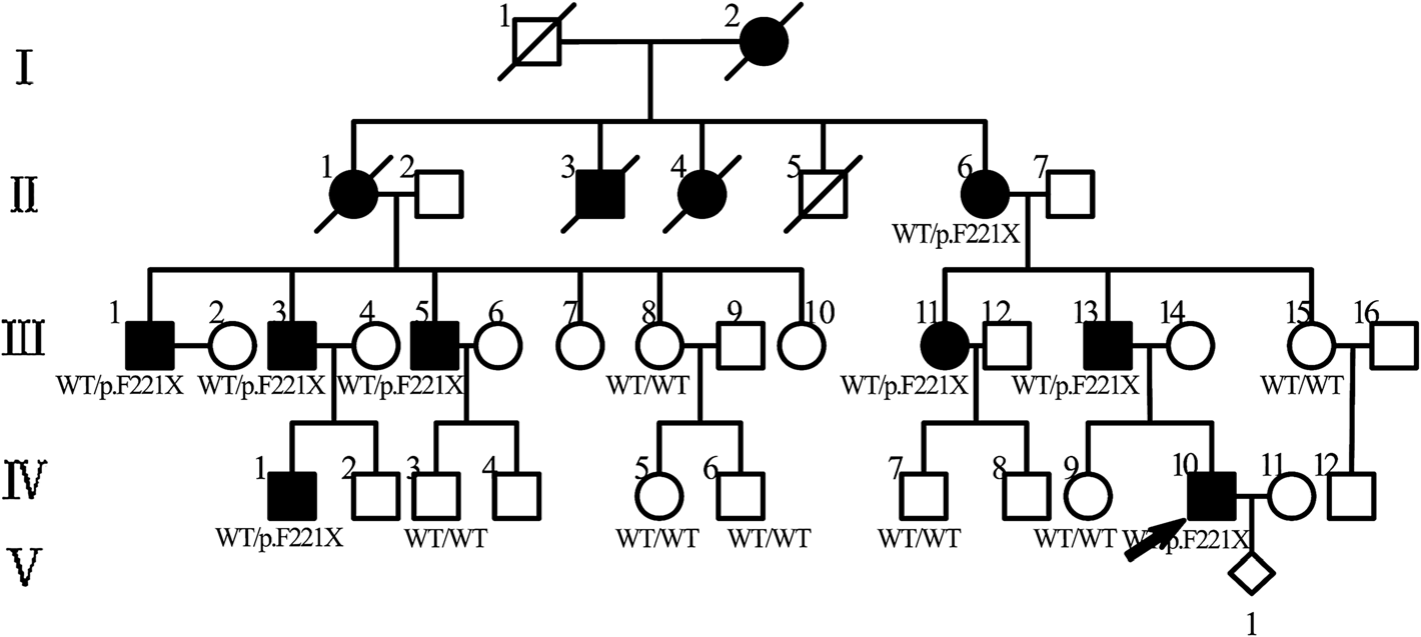
Pedigree diagrams of the family with autosomal dominant hearing loss (Open symbols, unaffected; solid symbols, affected; rhombus, fetus; symbols with dashed line, deceased; arrow indicates the proband).

A five-generation hearing loss-affected family of Han origin from Xinji city in Hebei Province was collected (Figure 2). In total 15 individuals, 8 affected and 7 unaffected members, participated. Detailed medical histories of the family members were obtained using a questionnaire regarding the following: subjective degree of hearing loss, age at onset, evolution of the hearing loss, presence of tinnitus and vertigo, history of head trauma and medication with aminoglycosides, noise exposure, pathological changes in the ear, goiter and other relevant clinical manifestations, family history, and the pregnancy/labor process. Physical examinations ruled out the probability of syndromic hearing loss. Since neither the deaf patients nor the normal hearing controls in the family reported cardiac discomfort, related examinations like ECG or ultrasound were not performed. Hearing was evaluated through otological examinations and audiological evaluations, including pure-tone audiometry (PTA), immittance, and auditory brainstem response (ABR). PTA was calculated as an average of the thresholds measured at 0.5, 1.0, 2.0, and 4.0 kHz. Air-conduction threshold measurements were performed at 125-8000 Hz in all participants, and bone-conduction thresholds were measured at 250-4000 Hz to check conductive hearing loss in affected individuals. The level of hearing loss was described as follows depending on PTA: normal hearing, below 20 dB, mild hearing impairment, 21–40 dB, moderate hearing impairment, 41–70 dB, severe hearing impairment, 71–95 dB, and profound hearing impairment, >95 dB. A high-resolution temporal bone CT scan was performed in affected individuals.

Genomic DNA from the 15 family members and 1000 normal controls was extracted from peripheral blood leukocytes using a commercially available DNA extraction kit (Qiagen, Hilden, Germany) according to the manufacturer’s instructions. Ultraviolet spectrophotometry was used to measure the DNA concentration and purity.

### Common deafness gene screening

Mutation screening was conducted using polymerase chain reaction (PCR) amplification and exons were sequenced directly. The common deafness genes included *GJB2*, *SLC26A4*, and the *mitochondrial 12S rRNA*, and the primers and PCR conditions for *GJB2, SLC26A4*, and the mitochondrial gene were described in detail in our previous paper [6].

### Targeted sequencing and variation analysis

Exons and the flanking 50 bp from 130 known human deafness genes were selected for capture and NGS sequencing using an Illumina HiSeq2000. Hybridization probes of 0.5 to 1.6 kilobase pairs (kbp) were generated for these genes from either cDNA clones of the genes or by PCR amplification from targeted gDNA regions. To ensure reliable capture of shorter exons, we specifically generated longer hybridization probes from gDNA for those exons that were shorter than 50 bp by including ~100-bp genomic DNA flanking the exons on both sides. All PCR products (10 ng each) were purified using the QIAquick PCR Purification Kit (Qiagen, Valencia, CA, USA) before use. Further details of the capture probe validation and preparation can be found in a previous report [7]. Fragmented gDNA libraries for Illumina GAII sequencing were prepared using the NEBNext DNA Sample Prep Master Mix set (E6040; New England Biolabs, Ipswich, MA, USA). End repair of DNA fragments, addition of a 3’ adenine (A), adaptor ligation, and reaction clean-up were performed according to the manufacturer’s protocol. The libraries were purified and size-selected using the AMPure DNA Purification kit (Beckman, Agencourt, Danvers, MA, USA). The ligated product (20 ng) was amplified over 14 PCR cycles using the Illumina PCR primers InPE1.0 and InPE2.0 and indexing primers according to the manufacturer’s instructions.

For the targeted enrichment of deafness genes, the Illumina library DNA was purified using a QIAquick MinElute column and eluted into 50- μL hybridization buffer (Roche, NimbleGen, Madison, WI, USA). The barcoded Illumina gDNA libraries (5 μg) were incubated in 16-μL hybridization buffer on the surface of hybridization glass slides on a hybridization station (BioMicro Systems, Inc., Salt Lake City, UT, USA) at 42°C for 72 h. Non-specific DNA fragments were removed after a series of six washing steps in washing buffer (Roche). The DNA bound to the probes was eluted by a 10-min incubation with NaOH (425 mL, 125 mM). The eluted solution was transferred to a 1.5-mL microcentrifuge tube containing 500-μL neutralization buffer (Qiagen’s PBI buffer). The neutralized DNA was desalted and concentrated on a QIAquick MinElute column and eluted into 30-μL EB buffer. To increase the yield, we typically amplified 5-μL eluted solution by 12 cycles of PCR using the Illumina PCR primers InpE1.0 and 2.0. Enrichment of the targeted deafness genes was examined by comparing the growth curves of captured and non-captured samples during quantitative PCR [7]. Twelve barcoded libraries of captured samples were pooled, and paired-end Illumina sequencing was performed using the Illumina HiSeq system (Illumina, San Diego, CA, USA). Details of the bioinformatics analysis methods used have been published previously [7].

### Sanger sequencing

Specific exons containing candidate variations were amplified by PCR using primer sets designed with the Primer3 software (http://www-genome.Wi.mit.edu/cgi-bin/primer/primer3_www.cgi). Each variation was sequenced by the Sanger sequencing method using an ABI 3730xl DNA sequencer (GENEWIZ Biosystems Corps. Beijing, China). Finally, accurate genotypes of the variations were confirmed by sequence analysis using the Sequencing Analysis software in family members.

## Results

### Clinical evaluations

**Figure 3:**
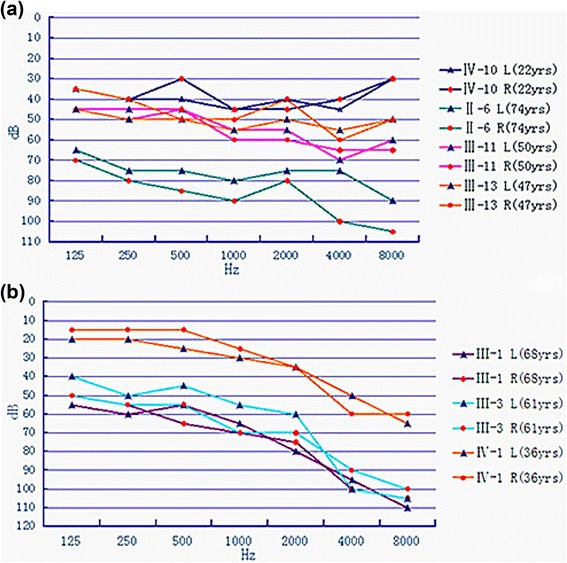
Tonal audiometric curves of the affected family members. The audiogram pattern of II-6 **(a)** branch affected all frequencies from the beginning, while the branch II-1**(b)** affected high frequencies initially, expanding to all frequencies at a later stage.

The family exhibited a typical autosomal dominant inheritance pattern of hearing loss (Figure 2). The reported onset of hearing impairment ranged from 20 to 40 years. The clinical history and audiological assessments of the eight affected members revealed a form of postlingual, bilateral, symmetrical, progressive, sensorineural hearing loss that affected both genders (Table 1). The audiogram patterns from the patients were distinct; in some cases, such as II-6, all frequencies were affected from the beginning. However, in other cases, such as II-1, high frequencies were affected initially, expanding to all frequencies at a later stage (Figure 3). Most of affected subjects had tinnitus and none had experienced vestibular dysfunction or other clinical abnormalities indicating syndromic hearing loss. DPOAE testing in all affected individuals showed cochlear dysfunction. Temporal bone CT scans of the affected members were normal.

**Table 1:** **Phenotypes and genotypes of the Chinese DFNA family**

**Member**	**Gender**	**Age (years)**	**Genotype**		**Phenotype**		
			**Exon8 (c.544_545insA)**	**Protein (p.F221X)**	**Age of Onset (years)**	**PTA (Left) (dB)**	**PTA (Right) (dB)**
II-6	F	74	+	+	20	profound	77.5
III-1	M	68	+	+	40	83.75	80
III-3	M	61	+	+	35	73.75	77.5
III-5	M	56	+	+	35	65	71.25
III-8	F	52	—	—	--	N	N
III-11	F	50	+	+	30	56.25	57.5
III-13	M	47	+	+	30	52.5	52.5
III-15	F	34	—	—	--	N	N
IV-1	M	36	+	+	32	36.25	28.75
IV-3	M	31	—	—	--	N	N
IV-5	F	39	—	—	--	N	N
IV-6	M	30	—	—	--	N	N
IV-7	M	25	—	—	--	N	N
IV-9	F	26	—	—	--	N	N
IV-10	M	22	+	+	22	40	32.5

(F: female, M:male, +: mutation, —: no mutation, N:normal).

### Candidate genes analyses

We analyzed the *GJB2, SLC26A4*, and *mitochondrial 12S rRNA* genes, including c.1494C > T and c.1555A > G. The results were all negative.

**Figure 4:**
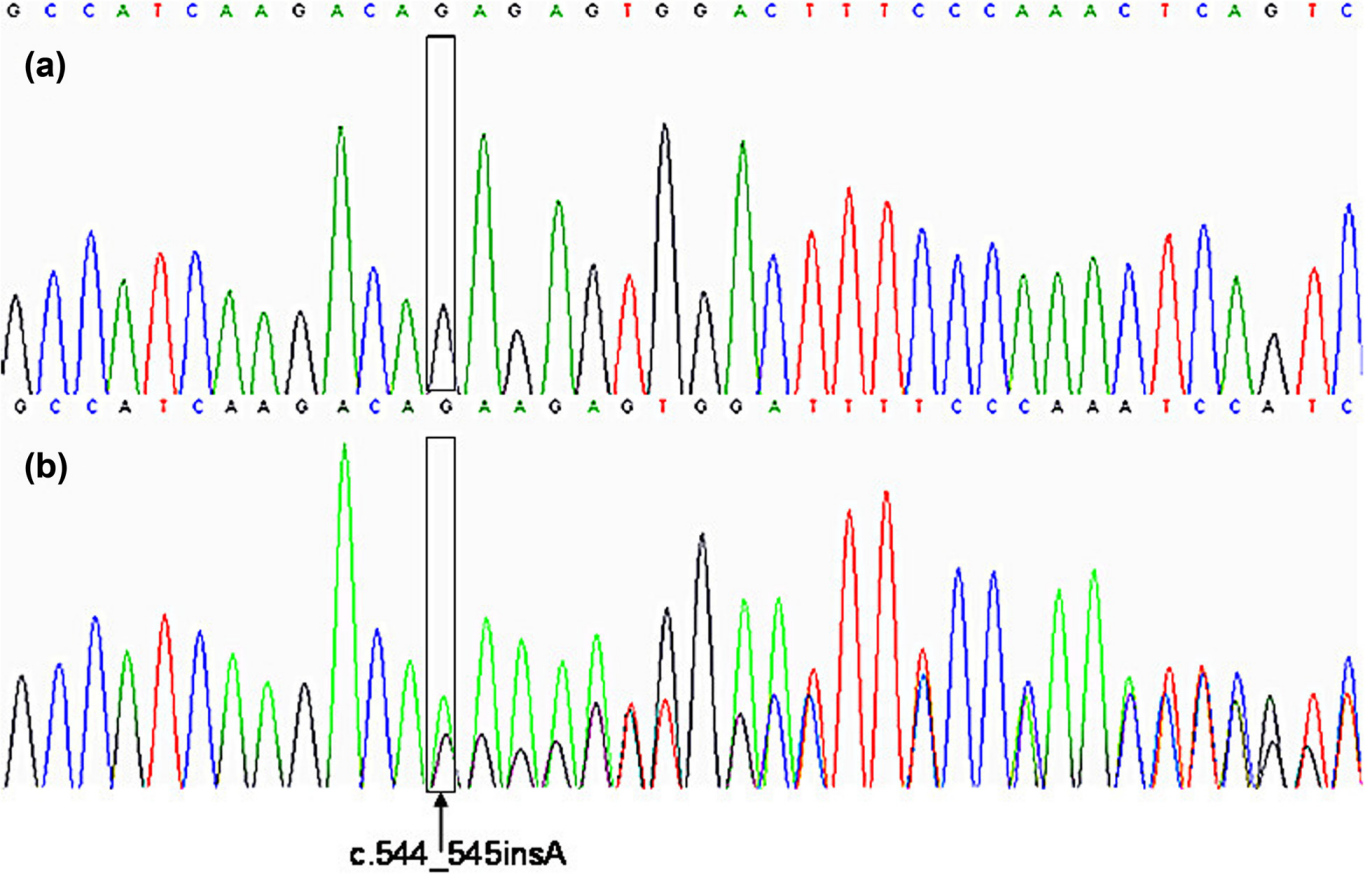
Part of the sequence of *EYA4* in exon 8. **(a)** Unaffected family member (III-15). **(b)** showing the heterozygote c.544_545insA mutation, the arrow indicates the site of insertion in trace. The box indicates the nucleobase difference between wild-type and the mutation.

The all 15 individuals were examined by NGS and sequenced by the Sanger sequencing method. Variants with known association with diseases, deleterious functional impact, and those with low PAF (<0.04) were selected as candidate mutations for further analysis and validation, and 20.4 ± 5.4 variants were obtained in each sample. In targeted sequencing, the c.544_545insA (p.F221X) mutation in the *EYA4* gene in exon 8 was identified in this family (Figure 4). This mutation was verified by Sanger sequencing. This mutation co-segregated with hearing loss in patients. The *EYA4* c.544_545insA (p.F221X) was analyzed in 1000 age-matched unrelated random controls with normal hearing and no carriers of the mutation were identified.

## Discussion

Connecting phenotype with genotype is the fundamental aim of genetics. The purpose of this study was to identify the genetic basis of hereditary hearing loss using NGS in the family and to provide genetic counseling to the family members. Using NGS, we identified a novel *EYA4* mutation, c.544_545insA, in this Chinese family with ADNSHL. The specific mutaiton, c.544_545insA in the *EYA4* was prioritised due to, 1) The mutaiton co-segregate with hearing loss in the family, 2) The mutation was verified by Sanger sequencing in all 15 members and the results are consistent with those of the NGS, 3) The specific mutation was analyzed in 1000 age-matched unrelated random controls with normal hearing and no carrier was identified.

*EYA4* is not a frequent gene in ADNSHL, versus other reported genes. To date, six families have been identified with ADNSHL linked to the DFNA10 locus (Figure 1 and Table 2). In 1996, O’Neill et al. [3] reported results of a linkage analysis in a large American family with ADNSHL, assigning the DFNA10 locus to a 15-cM interval on chromosome 6q22-23. By including distant branches of the family, they refined the size of the DFNA10 interval to a 6-cM region [8]. By further study, the group identified *EYA4* gene mutations in two unrelated families from Belgium and the USA segregating for deafness at this locus; they found different mutations in *EYA4*, c.1468insAA (p.342 fs) and c.2200C > T (p.R587X), both of which create premature stop codons [5]. A third truncating mutation of *EYA4* in a Hungarian family was revealed with an insertion of 4 bp (c.1558insTTTG). This insertion creates a frameshift and results in a stop codon at position 379 [9]. In 2007, another two new mutations in *EYA4* gene were identified. Makishima et al. [10] identified a novel frameshift mutation, c.1490insAA, of *EYA4* co-segregating with dominant hearing loss at the DFNA10 locus in an American family. The other was reported by Hildebrand et al. [11], a novel polypyrimidine tract variation, c.1282-12 T > A, that introduced a new 3’ splice acceptor site in intron 14. This was the first report of a point mutation in *EYA4* that was hypothesized to lead to aberrant pre-mRNA splicing and human disease. More recently, another *EYA4* novel mutation, p.I411K, was found by Tan M et al. [12].

**Table 2:** **Genotype and phenotype summary of known** ***EYA4*** **mutations**

**Family**	**Age of onset**	**Mutation**		**Protein domain**	**Exon**	**Tonal audiometric curves**	**Reference**
		**Amino acid**	**Nucleotide**				
America	10-50	c.1468insAA	p.342 fs	eyaHR	12	valley-flat	Wayne (2001) [3,5,8]
Belgium	0-40	c.2200C > T	p.R587X	eyaHR	20	valley-flat	Wayne (2001) [5]
Hungary	--	c.1558insTTTG	p.372 fs	eyaHR	13	valley-flat	Pfister (2002) [9]
America	10-50	c.1490insAA	p.349 fs	eyaHR	12	valley-flat	Makishima (2007) [10]
Australia	6-40	c.1282-12 T > A	p.426 fs	eyaHR	Before exon 15	valley-flat	Hildebrand (2007) [11]
Chinese	8-38	c.T1301A	p.I411k	eyaHR	15	valley-flat	Tan M (2014) [12]
Chinese	20-40	c.544_545insA	p.F221X	eyaHR	8	flat or sloping	Our study

The novel mutation we report here, c.544_545insA, in exon 8 in *EYA4*, like the previously characterized *EYA4* mutations, is predicted to affect the eyaHR domain, which is important post-developmentally for continued function of the mature organ of Corti [5,9]. The detected insertion of 1 bp creates a frameshift at position 182, resulting in a premature termination codon at position 221 (p.F221X). The effect is either a complete degradation of the mutant messenger or a nearly complete deletion of the eyaHR in the *EYA4* protein. We attempted to construct a three-dimensional structure for the mutant (p.F221X), but could not because the protein structure was affected severely. This may also suggest that the novel mutation is pathogenic rather than a polymorphism. From our and previous studies, frameshift mutations are common in the *EYA4* gene (Table 2).

The genotype-phenotype correlation of *EYA4* was analyzed. Most of the patients with missense mutations show bilateral, progressive, sensorineural hearing loss with onset beginning anywhere from prelingual to slightly above 50 years of age, even between members of the same family. At onset, hearing loss was mainly in the midfrequencies, with increasing age, all frequencies became affected. The hearing loss was initially mild, with a spontaneous evolution to a moderate or severe hearing impairment [13]. All total audiometric curves of these family members revealed valley-flat shape, also known as “cookie bite” curves. Our study shows affected individuals with ages of onset from 20 to 40 years, later than previous reports. The impairment initially affected high frequencies (II-1 branch) or all frequencies (II-6 branch), which differs from previous reports. Also, the audiograms of affected members are not characteristic cookie bite shape but flat or sloping. It is unclear whether this is related to a regional or ethnic/race origin difference, but this has not been reported in the previously published families.

In addition to our study, two Chinese ADNSHL families with *EYA4* mutations have been reported. In Tan’s [12] study, a missense mutation, p.I411K, was identified in *EYA4*. In that family, the onset of hearing loss was from 8 to 38 years, slightly earlier than our findings. At onset, hearing impairment was usually mild and detected in the mid-frequencies, resulting in an audiometric profile commonly referred to as a “cookie-bite” pattern. Along with progression, hearing loss began to involve other frequencies. All of these phenotypes were similar to the previous reports in other populations. Additionally, the reported ages of onset and the audiograms differed among branches of the family. In our study, the hearing loss age of onset and audiograms were differed in the two branches of the family. The audiogram pattern of the II-6 branch affected all frequencies from the beginning, and the onset age was 20–30 years, while branch II-1 affected high frequencies initially, expanding to all frequencies at a later stage, and the age of onset was later than in branch II-6, about 30–40 years. Whether these differences are due to environmental factors or potentially genes needs further study. We are currently following the hearing level of the next generation in the family and hope to provide them with more valuable genetic information*.*

## Conclusions

A novel *EYA4* mutation, c.544_545insA (p.F221X), was identified in a Chinese ADNSHL family using NGS and Sanger sequencing. The phenotype of the family is differs from the previous reported pedigrees with *EYA4* mutations in the age of onset and audiograms. The phenotypes also differed between individuals with the same mutation in the same family. Our results highlight the complexity of the *EYA4* genotype and phenotype.
